# Comparison of knowledge on asthma: doctors completing internal medicine residency and doctors completing medical school

**DOI:** 10.1590/S1516-31802001000300003

**Published:** 2001-05-02

**Authors:** Joaquim Edson Vieira, Alberto Cukier, Rafael Stelmach, David Itiro Kasahara, Silmar Gannam, Maria do Patrocínio Tenório Nunes Warth

**Keywords:** Medical Education, Residency, Internal medicine, Asthma, Educação médica, Residência, Clínica Médica, Asma

## Abstract

**CONTEXT:**

Asthma has been reported as a disease of increasing prevalence.

**OBJECTIVE:**

To assess the level of information and knowledge about asthma by means of a questionnaire among recent graduate physicians applying for medical residency at the Clinical Hospital of the University of São Paulo Medical School, Brazil.

**DESIGN:**

14 multiple-choice questions for asthma diagnosis and management.

**SETTING:**

University of São Paulo Medical School (FMUSP).

**PARTICIPANTS:**

Recent graduate physicians applying for the medical residency program at FMUSP in 1999 (n = 448) and physicians that had completed 2 year of internal medicine residency (n = 92).

**MAIN MEASUREMENTS:**

We applied a questionnaire with 14 multiple-choice questions about the management of asthma based upon the Expert Panel Report 2 – Guidelines for the Diagnosis and Management of Asthma, NIH/NHLBI, 1997 (EPR-2).

**RESULTS:**

The medical residency program in Internal Medicine improved treatment skills (the ability to propose adequate therapy) when compared to medical education (a score of 57.2% versus 46.9%, P < 0.001) but not diagnosis knowledge (understanding of asthma symptoms related to medicine intake) (33.5% versus 33.3%, P = 0.94). Treatment skills were higher among physicians who received their Medical Degree (MD) from public-sponsored medical schools in comparison with those from private schools [49.7 (SE 1.17)] versus [41.8 (SE 1.63)], P < 0.001.

**CONCLUSION:**

Medical schools might consider reevaluating their programs regarding asthma in order to improve medical assistance, especially when considering the general results for residents, as they were supposed to have achieved performance after completing this in-service training.

## RESUMO

**CONTEXTO:**

A asma vem sendo considerada uma doença de prevalência crescente.

**OBJETIVO:**

Investigar o grau de informação e conhecimento sobre asma utilizando um questionário aplicado para médicos recémgraduados, inscritos para o exame de residência médica, Hospital das Clínicas da Faculdade de Medicina da Universidade de São Paulo.

**TIPO DE ESTUDO:**

Questões de múltipla escolha sobre diagnóstico/tratamento de asma.

**PARTICIPANTES:**

Médicos recém-graduados (n = 448) inscritos para exame de residência médica no Hospital das Clínicas da Faculdade de Medicina da Universidade de São Paulo em 1999 e médicos que completaram dois anos de residência médica em clínica médica (n = 92).

**PROCEDIMENTOS:**

Um questionário com 14 questões de múltipla escolha sobre controle da asma baseado no manual editado: Expert Panel Report 2 – Guidelines for the Diagnosis and Management of Asthma, NIH/NHLBI, 1997 (EPR-2).

**RESULTADOS:**

Programa de residência médica em clínica médica melhora habilidades para tratamento (habilidade de propor terapia adequada, de acordo com EPR-2) quando comparado com graduação médica (57.2% versus 46.9%, P < 0, 001), mas não melhora a capacidade diagnóstica (entendimento dos sintomas de asma relacionado ao uso de medicamentos para seu controle) (33.5% versus 33.3%, P = 0, 94). Habilidades para tratamento foram melhores entre médicos que receberam sua formação em escolas médicas públicas quando comparados com médicos que receberam sua formação em escolas privadas [49.7 (SE 1.17)] versus [41.8 (SE 1.63), P < 0, 001].

**CONCLUSÃO:**

Escolas médicas devem considerar incrementar programas educacionais em asma, para estudantes e médicos residentes no Brasil, visando melhorar a assistência médica principalmente em se considerando residência médica, cuja performance poderia ser melhor, dado sua característica de treinamento em serviço.

## INTRODUCTION

Asthma has been reported as a disease of increasing prevalence. The diagnosis and management of asthma have standard guidelines, such as the Expert Panel Report 2 – Guidelines for the Diagnosis and Management of Asthma, Clinical Practice Guidelines, NIH/NHLBI, 1997 (EPR-2).^1^ This guideline reinforces the recommendations for anti-inflammatory medications for patients with moderate and severe asthma and further emphasizes the role of airway inflammation in the pathogenesis of this disease. Also, the asthma severity classification system has been updated to include the categories of mildly intermittent, mildly persistent, moderately persistent or severely persistent. With this system, anti-inflammatory medications are recommended for all patients bearing any persistent state. Despite the existence of such guidelines, the use of asthma medication related to the presence of symptoms has varied among countries or even between regions of one country.^2^

Health quality and the new healthcare environment – centered on patients – require certain competencies from the generalist physician. The knowledge and skills needed for continuous health improvement are in balance, and the best way to learn these are in a real context, learning the basics and focusing on the needs of the person assisted.^3^ It is to be expected that the knowledge of asthma would improve during medical education, which should be demonstrable by means of a questionnaire.^4^

A recent study using a questionnaire based on the National Heart, Lung and Blood Institute (NHLBI) guidelines showed an improvement during one year of training in a Pulmonary Diseases Program in relation to treatment, but not diagnosis of asthma, with 51% of medical residents giving answers that were less than 70% right.^5^ The clinical performance for primary care and internal medicine residents did not differ regarding management of chronic diseases, including asthma.^6^ These reports may suggest a need for more intensive teaching of diagnosis, treatment and control of asthma, even with the aid of specific programs developed during medical undergraduate and residency programs.

The prevalence of asthma symptoms among Brazilian children has been reported to be 15-30%.^7^ Although there are no clear Brazilian national statistics regarding adherence to the EPR-2 guidelines, a recent survey among pulmonary physicians from the Brazilian Society of Lung and Tuberculosis (Sociedade Brasileira de Pneumologia e Tisiologia) suggested that these guidelines, especially regarding management of asthma, need a more intensive and extensive distribution, in order to reach a greater number of physicians and to provide a better care profile.^8^ Indeed, by applying the International Asthma Guidelines, substantial success was achieved among low-income children from the city of São Paulo, removing patients from crisis-oriented management into a chronic care and preventive mode.^9^

There is a consensus that physicians must clearly understand guidelines in order to properly apply them. Also, it is expected that pulmonologists and allergy specialists are more likely to provide better care, complying with the asthma guidelines, than generalists.^10^ How-ever, our concern is that the generalist should have access to and better know an asthma guideline like the EPR-2, since he or she will be the first doctor to see a patient in any condition.

This study assessed the level of information and knowledge about asthma by means of a questionnaire among recent graduate physicians applying for medical residency in the year 2000, at the Clinical Hospital of the University of Sao Paulo Medical School, Brazil. This is the biggest residency program in Brazil. Entry to this program is awarded by means of a public examination, with nationwide applicants. Given the tradition of this institution, the exam is taken by candidates coming from all over the country, which may in some way give a picture of asthma training during medical education in Brazil. In fact, this sample represented approximately 7% of all medical graduates in the year 1999. Moreover, 60% of all medical graduates come from the southeast region, 23% from São Paulo State, and also the participation of students from states further south is low. The same questionnaire was applied after the completion of a 2-year residency program in Internal Medicine.

## METHODS

To evaluate the knowledge of asthma guidelines among recent MD graduates and after a 2-year internal medicine residency program, we used 14 multiple-choice questions adapted and translated into Portuguese from a questionnaire survey developed by Doerschug et al in 1999.^10^ None of the answers considered to be best by those authors were changed, and each question was designed to have a single correct answer. Internal medicine and pulmonary medicine physicians from our institution reviewed the questionnaire before given it to the examination takers.

The questionnaire was randomly applied to a sample of residency candidates (RC) right after they had finished their residency entrance test (n = 448 out of 2144). Medical specialty (R2) candidates that had finished their 2-year residency in Internal Medicine (n = 92) also participated in this survey. The residency entrance examination consisted of one hundred questions taken from the subjects of diagnosis (internal medicine, surgery, pediatrics, obstetrics/gynecology, and psychiatry), pathology, pharmacology, therapeutics, public health and epidemiology. We grouped these questions into the same core topics as in the questionnaire, i.e. dealing with diagnosis/assessment and therapeutics/pharmacology from general topics in medical sciences, in order to compare the two and to avoid any bias, since the questionnaire was taken after the examination.

The asthma questionnaire was divided into two major cores: diagnosis/assessment (questions 1, 8, 11, 24 and 25 from Doerschug, et al.) and treatment/pulmonary pharmacology (questions 2, 5, 6 13, 17, 18, 21, 22, and 31 from Doerschug, et al.). The first core of questions dealt with a classification of asthma from its symptoms, conditioning factors and pulmonary function test interpretation. The second core included the use of short and long-acting beta_2_-agonists and nebulizer devices, corticosteroid action, clinical indications for asthma medicines and their anti-inflammatory properties. Total scores and scores from each main core described above were calculated, and subjects were grouped into the RC and R2 groups.

All data were analyzed by Sigma-Stat statistical software. The RC and R2 groups were compared by using Student's t test [mean (standard error)]. The residency entrance examination questions (diagnosis/assessment and therapeutics/pharmacology) were compared by using Student's t test. P < 0.05 was considered significant.

## RESULTS

Medical residency candidates could be separated into two groups: those coming from public-sponsored medical schools, i.e. state and federal universities (289), and from private medical schools (159). Most of them achieved their medical degree graduation in the year 1999 (364) and 111 doctors graduated in 1998 or earlier.

Since the asthma questionnaire was taken right after the application of the medical residency entrance examination, no difference in performance was noted between the questionnaire and the entrance examination for the diagnosis/assessment and therapeutics/pharmacology answers (P = 0.377, t-test). This comparison was made in order to test for any bias between the examination and the questionnaire, with the two having the same structure in relation to such distribution.

**Figure 1:**
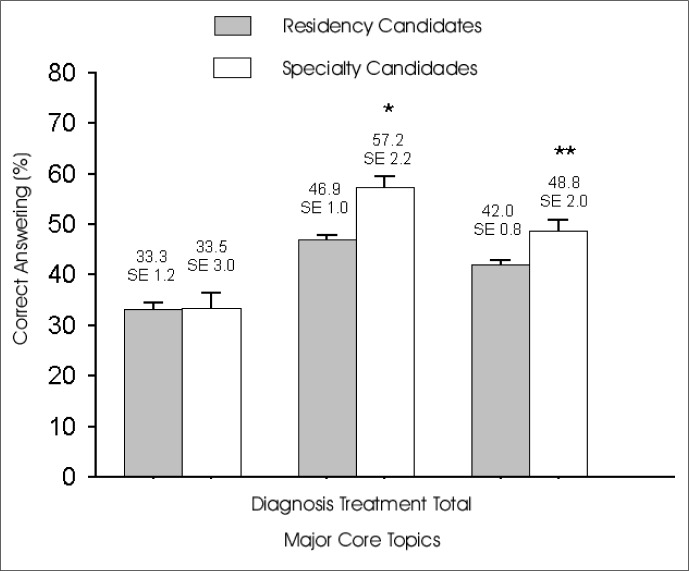
Percentages of correct answers to questions dealing with diagnosis, treatment and both, for Residency candidates (RC) and Specialty candidates (R2). *P < 0.001 and **P < 0.001, *t-*Student Test.

Residency candidates (RC) performed best in questions regarding asthma treatment, and lowest for those regarding diagnosis/classification. The percentage distribution for correct-answer scores among residency candidates was [33.26 (SE 1.16)] [mean (standard error)] for asthma diagnosis/classification, and [46.90 (SE 0.97)] for treatment/pulmonary pharmacology. The total score achieved was [42.03 (SE 0.79)]. Specialty candidates (R2) that had completed 2 years of the Internal Medicine program also performed best in questions regarding asthma treatment, presenting a performance for diagnosis/classification that was similar to RC. The percentage distribution for correct-answer scores for R2 was [33.48 (SE 3.01)] for asthma diagnosis/classification, [57.25 (SE 2.21)] for treatment/pulmonary pharmacology and [48.76 (SE 2.01)] for total score (Figure 1).

When comparing asthma diagnosis/classification knowledge between RC and R2 no significant difference was found (P = 0.94, t-Test), but for treatment skills R2 residents showed significantly higher skills (P < 0.001, t-Test). The total score was different when comparing these two groups (P < 0.001).

**Figure 2:**
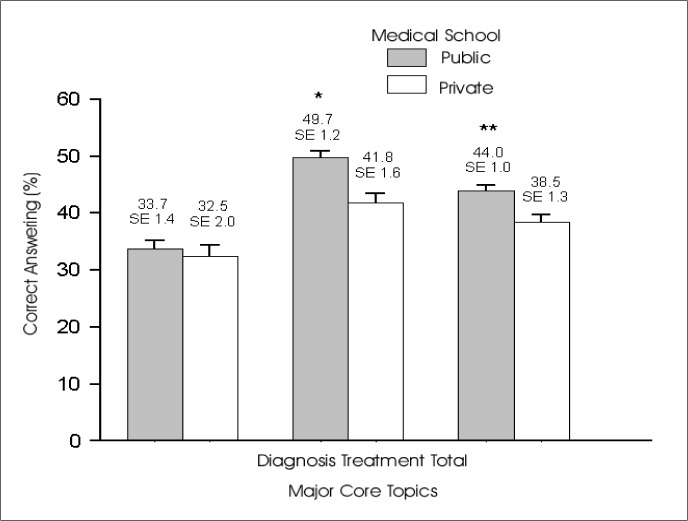
Percentage of correct answers to questions dealing with diagnosis, treatment and both, for Residency candidates that received their MD from public medical schools or private medical schools. *P < 0.001 and **P < 0.001, *t-*Student Test.

We also compared the performance of RC coming from government-sponsored schools and private ones. There was no difference for diagnosis/classification knowledge between those coming from public or private: [34.45 (SE 1.95)] versus [33.70 (SE 1.44)]. However, for treatment skills the public medical education system showed superiority: [49.71 (SE 1.17)] versus [41.78 (SE 1.63)], (P < 0.001, t-Test). The total score was different when comparing these two groups (P < 0.001) (Figure 2).

## DISCUSSION

The results from this study showed that a medical residency program in Internal Medicine undertaken over a two-year period improved asthma treatment skills but not diagnosis/classification knowledge, when comparing these competencies with those of recently graduated physicians (RC). In addition, the asthma diagnosis and treatment competence after completing medical education was very poor, with a total index of 42% for correct answers [5.39 (SE 3.56) out 14 questions]. Another interesting fact observed was that treatment skills were higher among those physicians who received their MD from public or Brazilian government-sponsored medical schools.

The intrinsic value of such questionnaires needs to be considered, as the questions used were important in giving an emphasis to the tied relationship between treatment recommendations and chronic disease severity. In this questionnaire, an incorrect answer may be understood as a deficiency in classifying the condition of a disease, and it is not intended to establish an actual competence in clinical diagnosis. However, underestimating a patient's condition would imply higher chances of mis-treatment. In this investigation, in addition to poor diagnosis or asthma classification skills, treatment attitudes can be considered barely appropriate. This may even suggest an attitude of low readiness to offer preventive practices.

The interpretation of the questionnaire mostly considers data related to clinical diagnosis. Complementary tests are not expected, but rather an interpretation of the physical diagnosis, mainly from the interview, which gives great consideration to medicine intake, which is a reliable source of information for interpreting EPR-2. The social or cultural background can cause some variations in providing such information, but we do not believe the recording of the answers to these questions might be affected by cultural or social bias from the medical staff.

The diagnosis, treatment and management of asthma as a chronic disease is essential for the success of programs set up to reduce its morbidity. Considering the increasing prevalence of asthma, such an approach can be life-saving. Despite the efforts to set up guidelines and make them clear, the compliance with such guidelines can still be considered distinct between different countries.^11^ This is probably because the introduction of new guidelines requires altering physician behavior, which is a very complex process.

This study has found that residency training positively influences abilities in asthma treatment. However, the knowledge of the classification system did not improve, even after an Internal Medicine program. The medical residency program is clearly an well-accepted way of learning and improving the physician's competence during in-training, hospital-based medical practice. Also, it is the time when the novelty is always questioned, given the experience of the staff, the great expectations with the new professional life and the obligatory board exams. So, it is the right time to present and improve guidelines, if they exist. The EPR-2 is a guideline accepted worldwide for the diagnosis and management of asthma as a chronic disease, but requires physician behavior to be updated.

Our results clearly show that the medical teaching system in Brazil has not been able to provide proper training in asthma knowledge and classification, including its management, according to EPR-2. This may arise from courses or clinical rounds in pulmonary and asthma management being too short, or even teacher deficiencies, given the low spread or acceptance of the guidelines.^12^ In Brazil, the medical course is provided as a 6-year undergraduate program: 2 basic, 2 pre-clinical and the last 2 consisting of clinical internship. This distribution, even taking in several aspects of many diseases, may not have been sufficient for leading students to the proper asthma classification expected by thorax and lung physician experts. Medical learning is a result of clinical training in services, reading and dedication, but good orientation and adequate models are essential in this education. Senior doctors must show medical students as many cases as necessary to make them better prepared to recognize and correctly treat asthma. Just knowing the lung sounds of such a disease may not be enough for understanding it and recognizing the human being hidden behind the disease.

We have not separated the RC into their diverse residency specialty options. Some may argue that physicians not interested in clinical specialties may show low readiness of knowledge about clinically related diseases. However, asthma is very common and we strongly believe both RC and R2 must have a good background in order to provide better control over this disease. Nonetheless, we should investigate these questions further, in another article.

The Internal Medicine residency program, in which young physicians receive their clinical training, did not improve their knowledge of the classification of asthma severity according to EPR-2 guidelines, although it improved their previous treatment skills. It could be due to the same reasons suggested above for medical students, or to an inadequate distribution of training time, which would be our guess. If this is correct, the clinical competence in residency needs to have an additional emphasis on classification as a means to properly treat this condition. Also, these findings support the need for continuing medical education related to asthma guidelines. This is strikingly important in Brazil, where residency is the final educational step for the majority of physicians beginning their professional life.

## CONCLUSION

Brazilian medical schools might consider applying internationally approved guidelines to their programs regarding asthma, thereby allowing better understanding of the asthma severity classification system and applying treatment paradigms based on disease activity. This educational profile may ensure higher chances of increased healthcare quality from medical students and residents in training, as a way of improving medical assistance in this country.
